# Development of software for measuring brain amyloid accumulation using ^18^F‐florbetapir PET and calculating global Centiloid scale and regional *Z*‐score values

**DOI:** 10.1002/brb3.3092

**Published:** 2023-06-07

**Authors:** Hiroshi Matsuda, Tsutomu Soma, Kyoji Okita, Yoko Shigemoto, Noriko Sato

**Affiliations:** ^1^ Drug Discovery and Cyclotron Research Center Southern Tohoku Research Institute for Neuroscience Koriyama Japan; ^2^ Department of Radiology National Center of Neurology and Psychiatry Kodaira Japan; ^3^ Software Development Department PDRadiopharma Inc. Tokyo Japan; ^4^ Department of Nuclear Medicine and Medical Physics International University of Health and Welfare School of Medicine Narita Japan; ^5^ Department of Psychiatry National Center of Neurology and Psychiatry Kodaira Japan

**Keywords:** 18F‐florbetapir, amyloid, Centiloid scale, PET

## Abstract

**Background and purpose:**

Quantitative measures have been proposed to aid the visual interpretation of amyloid PET. Our objective was to develop and validate quantitative software that enables calculation of the Centiloid (CL) scale and *Z*‐score for amyloid PET with ^18^F‐florbetapir.

**Methods:**

This software was developed as a toolbox in statistical parametric mapping 12 running on MATLAB Runtime. For each participant's amyloid PET, this software calculates the CL scale using the standard MRI‐guided pipeline proposed by the Global Alzheimer's Association Interactive Network (GAAIN) and generates a *Z*‐score map for comparison with a new amyloid‐negative database constructed from 20 healthy controls. In 23 cognitively impaired patients with suspected Alzheimer's disease, *Z*‐score values for a target cortical area from the new database were compared with those from the GAAIN database constructed from 13 healthy controls. The CL values obtained using low‐dose CT of PET/CT equipment were then compared with those obtained using MRI.

**Results:**

The CL calculation was validated with the ^18^F‐florbetapir dataset in the GAAIN repository. *Z*‐score values obtained from the new database were significantly higher (mean ± standard deviation, 1.05 ± 0.77; *p* < .0001) than those obtained from the GAAIN database. The use of low‐dose CT provided CL scales that were highly correlated with those obtained with MRI (*R* ^2^ = .992) but showed a slight yet significant underestimation (−2.1 ± 4.2; *p* = .013).

**Conclusions:**

Our quantification software provides the CL scale and *Z*‐score for measuring overall and local amyloid accumulation with the use of MRI or low‐dose CT.

## Graphical abstract

**Development of quantitative software for amyloid PET with 18F‐florbetapir Calculation of global Centiloid scale and regional Z‐score values for measuring brain amyloid accmulation**

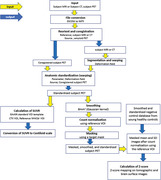

## 1 INTRODUCTION

The clinical impact of amyloid PET on the diagnosis and patient management of Alzheimer's disease (AD) has been reported in many studies (Boccardi et al., 2016; Ceccaldi et al., 2018; Matsuda et al., 2022; Rabinovici et al., 2019; Zwan et al., 2017). In our recent multicenter study (Matsuda et al., 2022) using ^18^F‐florbetapir, amyloid PET results substantially changed the pre‐scan AD/non‐AD diagnosis and patient management plans in 39% and 42% of patients, respectively. These changes were driven by visual interpretation of the positivity/negativity of amyloid PET. However, when amyloid accumulation is low, this dichotomized visual interpretation tends to vary among readers. For ^18^F‐florbetapir PET, the κ coefficient, which indicates inter‐reader reliability, is high but ranges from just 0.69 to 0.74 (Camus et al., 2012; Matsuda et al., 2022; Nayate et al., 2015).

To aid the visual interpretation, quantitative measures of amyloid accumulation in the brain have been proposed. In particular, the Centiloid (CL) (Klunk et al., 2015) scale has become widely used in recent years as a harmonized value for standardizing each analytical method or PET ligand used. Several studies have reported CL thresholds for amyloid positivity. For example, comparative studies between antemortem PET and postmortem neuropathology reported that a CL less than 10 can exclude AD due to the absence of neuritic plaques (Amadoru et al., 2020) and that a cutoff of 12.2 CL detects moderate‐to‐frequent neuritic plaques (La Joie et al., 2019). Positive visual interpretations are reported to be highly consistent for CL scales of 26 and above (Amadoru et al., 2020; Matsuda et al., 2022), whereas CL scales from 12 to 30 are often equivocal findings on visual evaluation and are referred to as the gray zone (Milà‐Alomà et al., 3021; Pemberton et al., 2022). As an adjunct to the visual detection of focal early amyloid accumulation in this gray zone, a comparison with a database of amyloid‐negative controls, rather than with the whole brain CL scale alone, may be helpful (Lilja et al., 2016). We have already developed software, called Amyquant (Matsuda & Yamao, 2022), that can automatically compute the CL scale and calculate a *Z*‐score by comparing a patient's PET to a database constructed from healthy controls in the Global Alzheimer's Association Interactive Network (GAAIN) repository (http://www.gaain.org/centiloid‐project). Because the number of healthy controls for ^18^F‐florbetapir in Amyquant is just 13, it would be desirable to collect more healthy controls to improve the accuracy of the *Z*‐score analysis.

Accordingly, the purpose of the present study was to develop new quantitative software for amyloid PET with ^18^F‐florbetapir that enables the CL scale calculation and *Z*‐score analysis as a comparison with a database constructed from a larger number of healthy controls. Although MRI is used to anatomically standardize PET for the calculation of the CL scale, another study purpose was to determine whether low‐dose CT for attenuation correction used in PET/CT equipment can be a substitute for MRI. We have already confirmed the substitutability of low‐dose CT in the calculation of the CL scale with ^18^F‐flutemetamol PET (Matsuda et al., 2021) and, in this study, we examine its application to ^18^F‐florbetapir.

## 2 METHODS

### 2.1 Participants

The participants were 23 patients (14 women and 9 men; 75.4 ± 7.4 years old, range, 48–82 years) and 20 cognitively healthy adults (13 men and 7 women; 45.4 ± 3.9 years old, range, 35–50 years) enrolled in a previous multicenter study (Matsuda et al., 2022). The patients were recruited from an outpatient memory clinic of the National Center of Neurology and Psychiatry, Japan. They had a mini–mental state examination (MMSE) (Folstein et al., 1975) score of 23.8 ± 2.6 (range, 20–29). According to National Institute on Aging and the Alzheimer's Association criteria (McKhann et al., 2011), of the 23 patients, 10 and 13 were diagnosed as having possible and probable AD, respectively. Cognitively healthy adults with an MMSE exceeding 29 were studied to construct an amyloid‐negative database.

This study was approved by the certified Clinical Research Review Board at the National Center of Neurology and Psychiatry and was registered in the Japan Registry of Clinical Trials (jRCTs031180446). Written informed consent was obtained for all participants or their legal representatives.

### 2.2 Image acquisition

#### 2.2.1 ^18^F‐florbetapir PET/CT

Each participant received an intravenous injection of 389 ± 11 (range, 365–403) MBq of ^18^F‐florbetapir (Amyvid, PDRadiopharma Inc., Tokyo, Japan). All PET acquisitions were performed using a hybrid PET/CT Biograph 16 True‐point scanner (Siemens Healthineers, Erlangen, Germany). After patient positioning, a low‐dose CT scan (kVp, 130 KeV; current, 40 mA; rotation time, 1.0 s; table feed per rotation, 7.2 mm; spiral pitch factor, 0.75) was acquired to be used for attenuation correction of the PET data. CT images were reconstructed using the “H10s very smooth” kernel, a 30.0‐cm reconstruction field of view, and a 2.0‐mm slice interval, which gave a voxel size of 0.59 × 0.59 × 2.0 mm^3^. This low‐dose CT protocol delivers head radiation doses of 0.4 mSv. A three‐dimensional (3D)‐PET acquisition with list mode was started from 40.6 ± 1.5 (range, 39–44) min after the injection of ^18^F‐florbetapir and lasted for 20 min. In the present study, 10 min acquisition data from 50.6 ± 1.5 (range, 49–54) min were used for calculation of the CL scale because the equation for converting the standardized uptake value ratio (SUVR) to the CL scale was derived from 10 min data acquisition from 50 min postinjection (Navitsky et al., 2018). Image reconstruction was performed using a 3D ordered‐subset expectation maximization algorithm with the following parameters: image matrix, 168; field of view, 300 mm; subsets, 21; iterations, 4; post‐filter (Gaussian), 4‐mm full width at half maximum (FWHM); attenuation correction, CT‐guided. The resulting voxel size was 2.02 × 2.06 × 2.03 mm^3^.

#### 2.2.2 MRI

The MRI for all patients was performed on an Achieva 3.0‐T MR scanner (Philips Medical Systems, Best, The Netherlands) equipped with a 32‐channel coil within 38 ± 25 (range, 1–81) days before amyloid PET. 3D T1‐weighed MRI (3DT1WI) was acquired for each participant using a volumetric turbo field echo T1‐weighted structural sequence (300 sagittal slices; repetition time, 7.0 ms; echo time, 3.4 ms; field of view, 260 × 240 mm; voxel size, 0.7 × 0.7 × 0.6 mm^3^; flip angle, 10°).

### 2.3 Processing pipeline of the software

**FIGURE 1:**
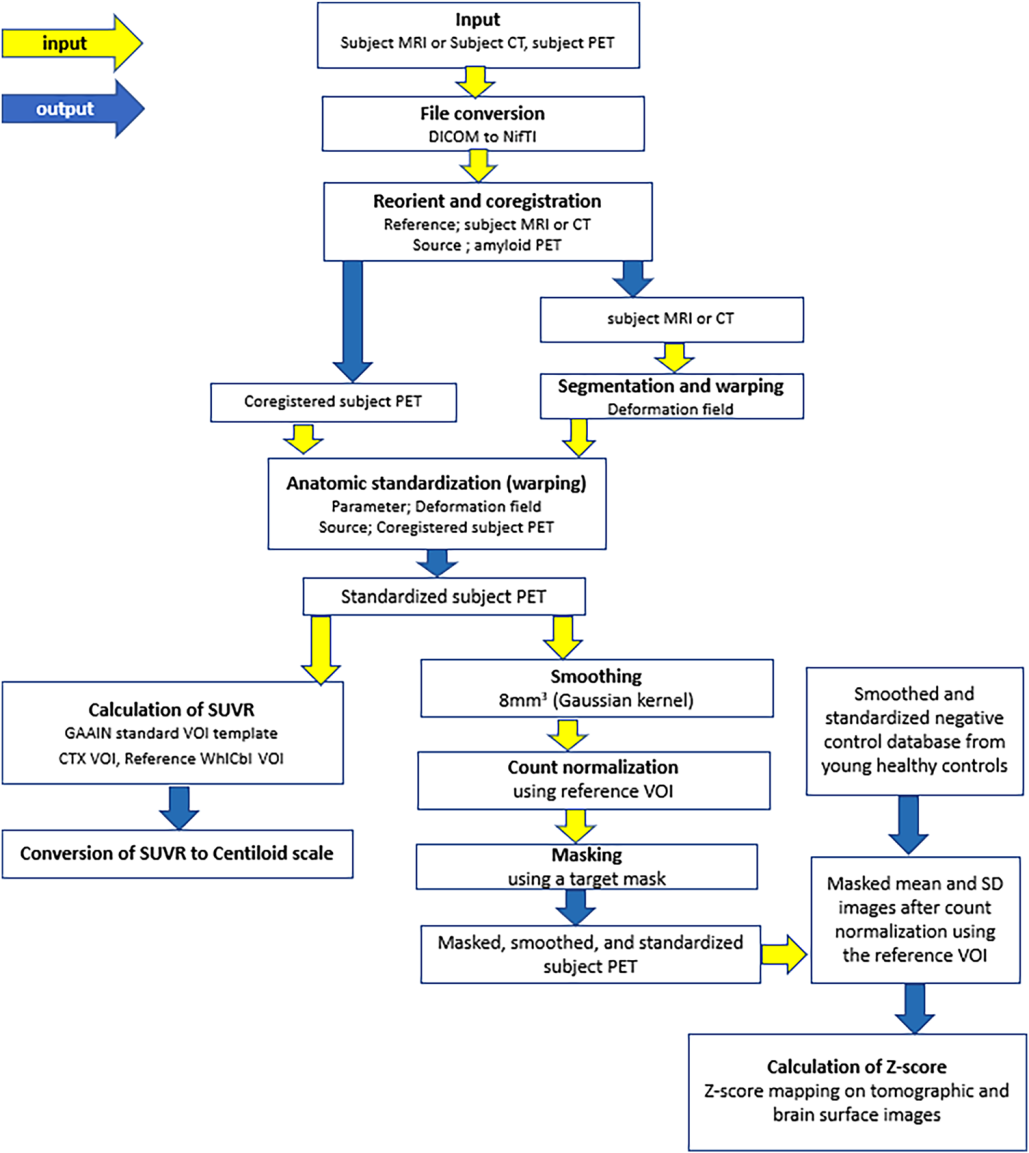
Processing pipeline for the software for quantifying amyloid accumulation by ^18^F‐florbetapir PET one process entails calculation of the Centiloid (CL) scale from each participant's amyloid PET and MRI or CT. Another process involves the statistical comparison of each participant's amyloid PET with a database constructed from negative amyloid PET results obtained from healthy controls. These two processes are automatically executed sequentially as an SPM12 toolbox running in MATLAB Runtime on the Windows operating system.

The present software, named AMYclz, was developed as a toolbox in statistical parametric mapping (SPM, https://www.fil.ion.ucl.ac.uk/spm) 12 running on MATLAB Runtime on the Windows operating system. This software involves two distinct processes: calculation of the CL scale from each participant's amyloid PET and MRI or CT, and statistical comparison of each participant's amyloid PET with a database constructed from amyloid‐negative PET results obtained from 20 healthy controls (Figure 1). This software requires about 4 min to complete all of the steps for a single participant using a 64‐bit laptop PC (CPU, Intel Core i7, 2.60 GHz; memory, 16 GB).

The first process for quantitative analysis uses the SUVR and a 100‐point scale called the CL scale. First, a pair of PET and MRI images for each participant is input in DICOM or NIfTI format. Then, the MRI or CT and PET images are reoriented, and the reoriented PET images are coregistered to the MRI images. Next, the MRI or CT images are warped into Montreal Neurologic Institute (MNI) space using unified segmentation in SPM12. The parameters of the deformation field in this warping are applied to the coregistered PET images for anatomic standardization into MNI space. Using the standard volume of interest (VOI) in GAAIN, the SUVR was calculated from ^18^F‐florbetapir PET counts in the global cortical target area (GAAIN, CTX VOI) and in the whole cerebellum (GAAIN, WhlCbl VOI) as the reference area. Then, a direct conversion equation (CL = 175.2 × SUVR − 182.2) was applied to convert the SUVR to the CL value, as described previously (Navitsky et al., 2018). The CL scales obtained using MRI and CT for anatomic standardization were defined as CL_MRI_ and CL_CT_, respectively.

The second process involves the comparison of each participant's PET data with a negative control database constructed from healthy controls. The standardized PET images are smoothed using an 8‐mm (Ceccaldi et al., 2018) Gaussian kernel. The smoothed and standardized PET images are then masked to remove white matter areas with high counts after normalization of the PET count using a reference VOI count. Masked mean and standard deviation PET images are generated from an amyloid‐negative control database comprising smoothed and standardized PET images of healthy controls. A *Z*‐score map is displayed by overlay on tomographic sections with a contour of the target cortical VOI and with surface rendering of the standardized brain MRI using the following equation: *Z*‐score = ([individual count] − [mean count of control database])/(standard deviation count of control database). In the *Z*‐score mapping display, we can change the upper and lower *Z*‐score levels and the cluster size threshold.

### 2.4 Validation of the present software for CL calculation

The CL scales calculated using AMYclz based on the standard MRI‐guided pipeline were compared with the CL scales published on the GAAIN website for ^18^F‐florbetapir with a reference VOI of the whole cerebellum. This pipeline was validated with 46 pairs of ^18^F‐florbetapir PET and corresponding 3DT1WI datasets from 13 healthy controls (7 women and 6 men; 27.0 ± 4.3 years old, range, 21–35, MMSE above 29), 6 elderly healthy controls (4 men and 2 women; 63.1 ± 8.1 years old, range, 51–75, MMSE above 27), 3 at‐risk elderly individuals (3 men; 79.6 ± 2.9 years old, range, 78–83, MMSE above 28), 17 AD patients (9 women and 8 men; 67.0 ± 7.1 years old, range, 51–76, MMSE 21.8 ± 5.4), and 7 patients with mild cognitive impairment (6 men; 80.3 ± 9.2 years old, range, 64–89, MMSE 26.5 ± 1.4) downloaded from the GAAIN website.

### 2.5 Evaluation of *Z*‐score values using a newly constructed database from healthy controls

The mean positive *Z*‐score of the target cortical VOI obtained from AMYclz analysis based on the standard MRI pipeline in 23 patients was compared between that obtained using a newly constructed control database comprising 20 healthy controls and that obtained using a GAAIN database comprising 13 healthy controls. The mean positive *Z*‐score values obtained using the new database and the GAAIN database were defined as *Z*_new_db_ and *Z*_GAAIN_db_, respectively. In addition, SUVR values at the target cortical VOI were compared between the new database and the GAAIN database.

### 2.6 Evaluation of calculated CL scales and *Z*‐score values using low‐dose CT in patients

The CL scales calculated using AMYclz with the CT‐guided pipeline were compared with those obtained using the MRI‐guided pipeline in patients. The mean positive *Z*‐score values of the target cortical VOI obtained from the new database were also compared between MRI‐guided and CT‐guided pipelines in patients. The CL scales and mean positive *Z*‐score values obtained using MRI‐guided and CT‐guided pipelines were defined as CL_MRI_, CL_CT_, *Z*_MRI_, and *Z*_CT_, respectively.

### 2.7 Statistical analysis

Concordances between *Z*_new_db_ and *Z*_GAAIN_db_, between CL_MRI_ and CL_CT_, and between *Z*_MRI_ and *Z*_CT_ were assessed using Pearson correlation estimates and Bland–Altman plots. In the Bland–Altman plot, we performed a Spearman correlation to test whether there were associations between the difference and the load. CL scales and *Z*‐score values and their standard deviations were computed with mean absolute differences and limits of agreement. These statistical tests were performed using JMP ver. 16.2 (SAS Institute).

In addition, to investigate regional differences in the CT‐guided and MRI‐guided standardized amyloid PET images, a paired t‐test was applied to these images on a voxel basis after they were smoothed with an 8‐mm FWHM Gaussian kernel using SPM12. Results were considered significant with an extent threshold of 300 voxels corrected for multiple comparisons (family‐wise error [Flandin & Friston, 2019], *p* < .05).

## 3 RESULTS

### 3.1 Validation of the present software for CL calculation

Validation of the present processing pipeline using 46 pairs of ^18^F‐florbetapir PET and corresponding 3DT1WI datasets in the GAAIN repository indicated an excellent correlation with published data. The slope of the linear correlation was 1.0, with an intercept of 0.293, and the *R* ^2^ was .997, which were within the validation criteria defined by Klunk et al. (2015). These criteria state that the slope should be between 0.98 and 1.02 and the intercept between −2 and +2 CL for a linear regression equation and that the *R* ^2^ correlation coefficient should exceed 0.98. This validation of the present pipeline allowed use of the previously published equation (Navitsky et al., 2018) for the direct conversion of the ^18^F‐florbetapir SUVR to CL.

### 3.2 Evaluation of *Z*‐score mapping using a newly constructed database of cognitively healthy controls

**FIGURE 2:**
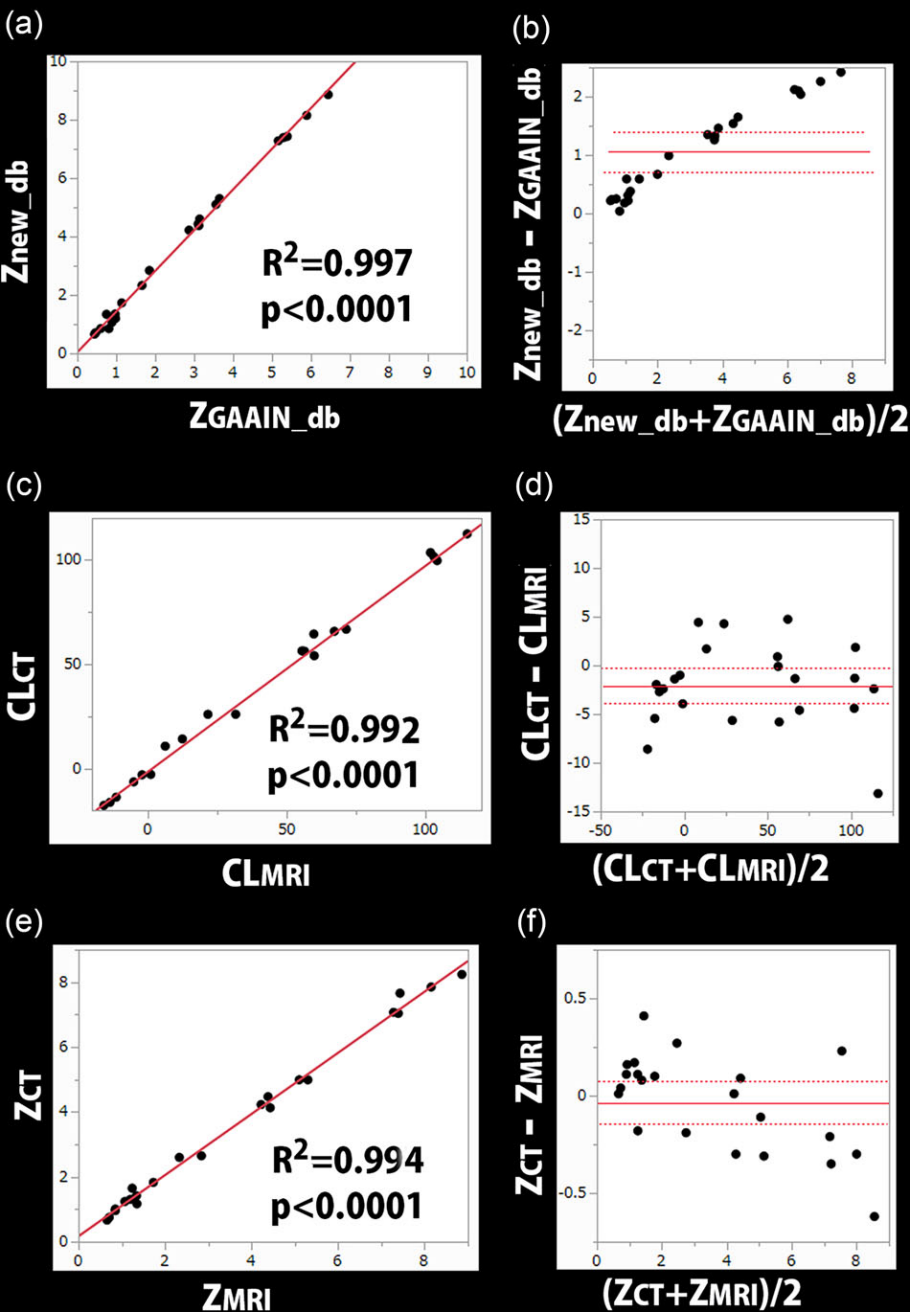
Comparison of the Centiloid (CL) scale and *Z*‐score values between different databases and between MRI‐guided and CT‐guided pipelines. Pearson correlation analysis (a, c, e) showed highly significant correlations (*p* < .0001) of *R* ^2^ = .997 between *Z*_new_db_ and *Z*_GAAIN_db_, of *R* ^2^ = .992 between CL_CT_ and CL_MRI_, and of *R* ^2^ = .994 between *Z*_CT_ and *Z*_MRI_. A Bland–Altman plot (b, d, f) showed that *Z*_new_db_ was significantly higher than *Z*_GAAIN_db_ (mean ± standard deviation, 1.05 ± 0.77; *p* < .0001), that CL_CT_ was slightly but significantly underestimated (−2.1 ± 4.2; *p* = .013) compared with CL_MRI_, and that the positive mean *Z*‐score for the target cortical volume of interest (VOI) was not significantly different between *Z*_CT_ and *Z*_MRI_ (*p* = .253). Spearman correlation analysis revealed a significant association between the difference in *Z*_new_db_ versus *Z*_GAAIN_db_ and *Z* load (*ρ* = .955, *p* < .0001), no significant association between the difference in CL_CT_ versus CL_MRI_ and CL load (*ρ* = −.083, *p* = .701), and a significant association between the difference in *Z*_CT_ versus *Z*_MRI_ and *Z* load (*ρ* = −.460, *p* = .027).

**FIGURE 3:**
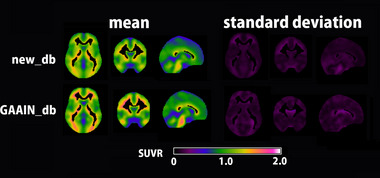
Standardized uptake value ratio (SUVR) images for the mean and standard deviation of the present new database and the Global Alzheimer's Association Interactive Network (GAAIN) database. In the target cortical volume of interest (VOI), the SUVR values (0.986 ± 0.052) for the present new database (new_db, 13 men and 7 women; 45.4 ± 3.9 years old) are significantly (*p* < .005) lower than those (1.048 ± 0.064) for the GAAIN database (GAAIN_db, 7 women and 6 men; 27.0 ± 4.3 years old).

**FIGURE 4:**
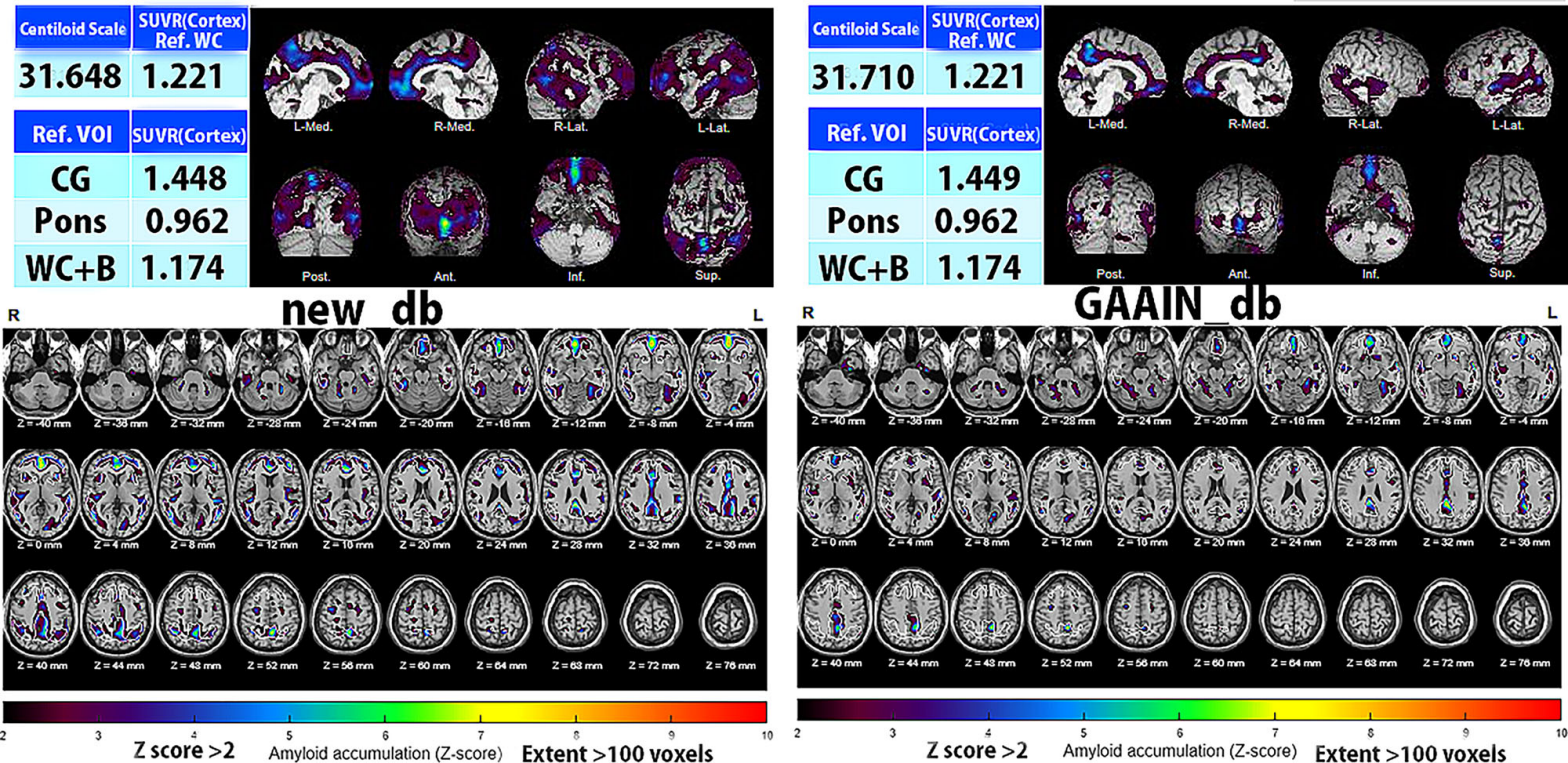
Comparison of analytical results by AMYclz between the new database and Global Alzheimer's Association Interactive Network (GAAIN) database. A *Z*‐score map is displayed by overlay on tomographic sections with a contour of the target cortical volume of interest (VOI) and with a surface rendering of the standardized brain MRI. A regionally higher *Z*‐score was obtained from the present new database (left) than from the GAAIN database (right).

Pearson correlation analysis revealed a highly significant correlation of *R* ^2^ = .997 between *Z*_new_db_ and *Z*_GAAIN_db_ (*p* < .0001, Figure 2a). The linear regression equation was *Z*_new_db_ = 1.393 × *Z*_GAAIN_db_ + 0.04. A Bland–Altman plot showed that *Z*_new_db_ was significantly higher than *Z*_GAAIN_db_ (mean ± standard deviation, 1.05 ± 0.77; *p* < .0001, Figure 2b). The 95% limits of agreement ranged from 0.7 to 1.4. Spearman correlation analysis revealed a significant association between the difference in *Z*_new_db_ versus *Z*_GAAIN_db_ and *Z* load (*ρ* = .955, *p* < .0001). The mean and standard deviation images for the new database and GAAIN database are demonstrated in Figure 3. SUVR values in the target cortical VOI were significantly lower in the new database (0.986 ± 0.052) than in the GAAIN database (1.048 ± 0.064) (*p* < .005). Results of AMYclz analysis using the new database and GAAIN database are presented for a representative case (Figure 4).

### 3.3 Evaluation of the calculated CL scales and *Z*‐score using low‐dose CT in patients

**FIGURE 5:**
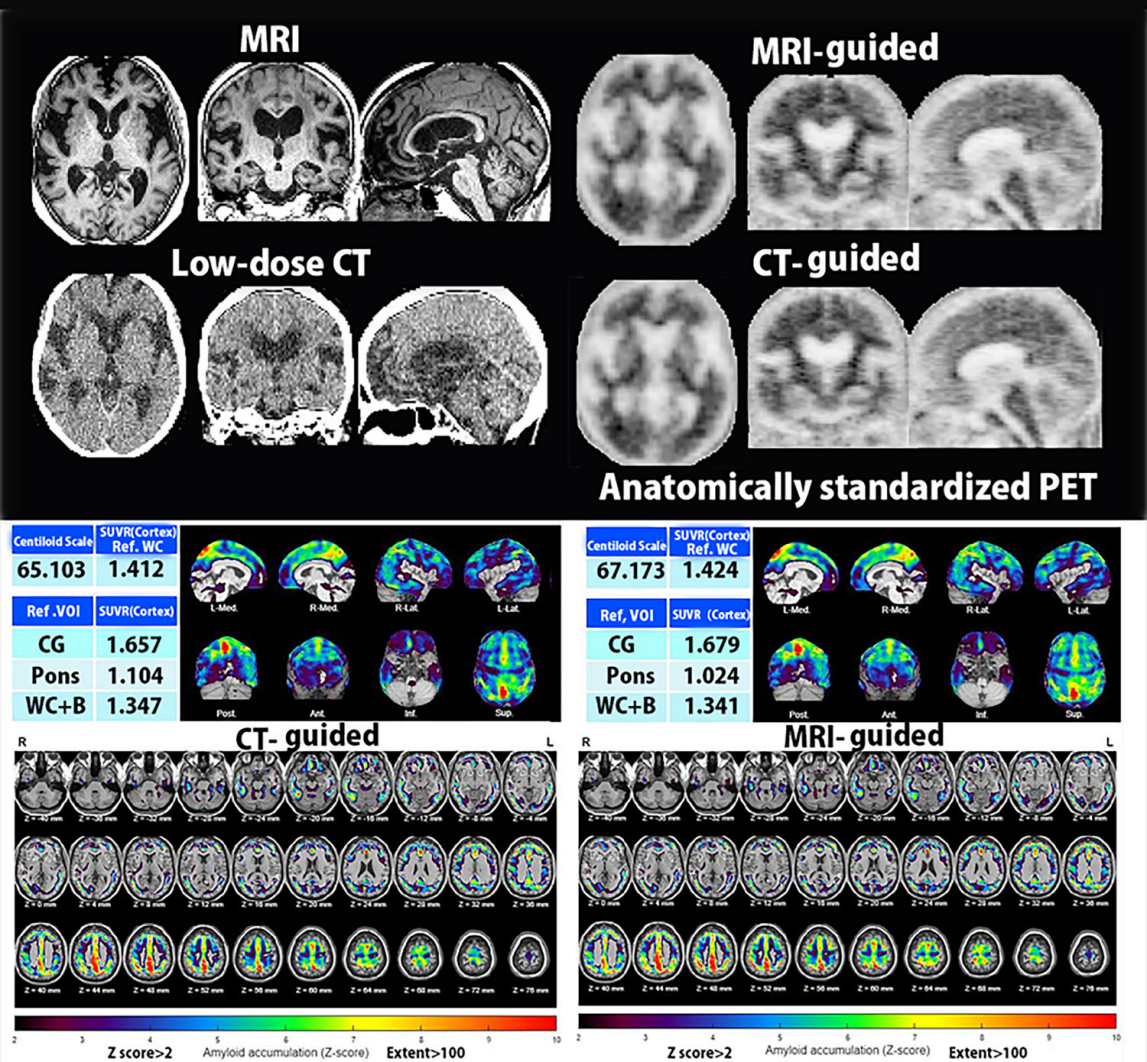
Comparison of analysis results from AMYclz CT‐guided and MRI‐guided pipelines (top). Anatomically standardized MRI, low‐dose CT, and amyloid PET images are shown; MRI‐guided and CT‐guided anatomical standardization resulted in nearly identical PET images. (Bottom) Results of AMYclz analysis of CT‐guided and MRI‐guided pipelines are shown; the CT‐guided pipeline (left) yielded a slightly lower CL65.1 than the CL67.1 obtained for the MRI‐guided pipeline (right), but the *Z*‐score maps were identical for these two pipelines.

MRI‐guided and CT‐guided anatomically standardized PET images (Figure 5) are presented, along with results of AMYclz analysis, for a representative case. Pearson correlation analysis showed a highly significant correlation of *R* ^2^ = .992 between CL_CT_ and CL_MRI_ (*p* < .0001, Figure 2c). The linear regression equation was CL_CT_ = 0.989 × CL_MRI_ − 1.68. A Bland–Altman plot showed that CL_CT_ was slightly but significantly underestimated (mean ± standard deviation, −2.1 ± 4.2; *p* = .013) compared with CL_MRI_ (Figure 2d). The 95% limits of agreement ranged from −3.9 to −0.3. Spearman correlation analysis did not reveal a significant association between the difference in CL_CT_ versus CL_MRI_ and CL load (*ρ* = −.083, *p* = .701).

Pearson correlation analysis revealed a highly significant correlation of *R* ^2^ = .994 between *Z*_CT_ and *Z*_MRI_ (*p* < .0001, Figure 2e). A Bland–Altman plot showed that the positive mean *Z*‐score for the target cortical VOI was not significantly different between *Z*_CT_ and *Z*_MRI_ (*p* = .253, Figure 2f). Spearman correlation analysis identified a significant association between the difference in *Z*_CT_ versus *Z*_MRI_ and *Z* load (*ρ* = −.460, *p* = .027).

**FIGURE 6:**
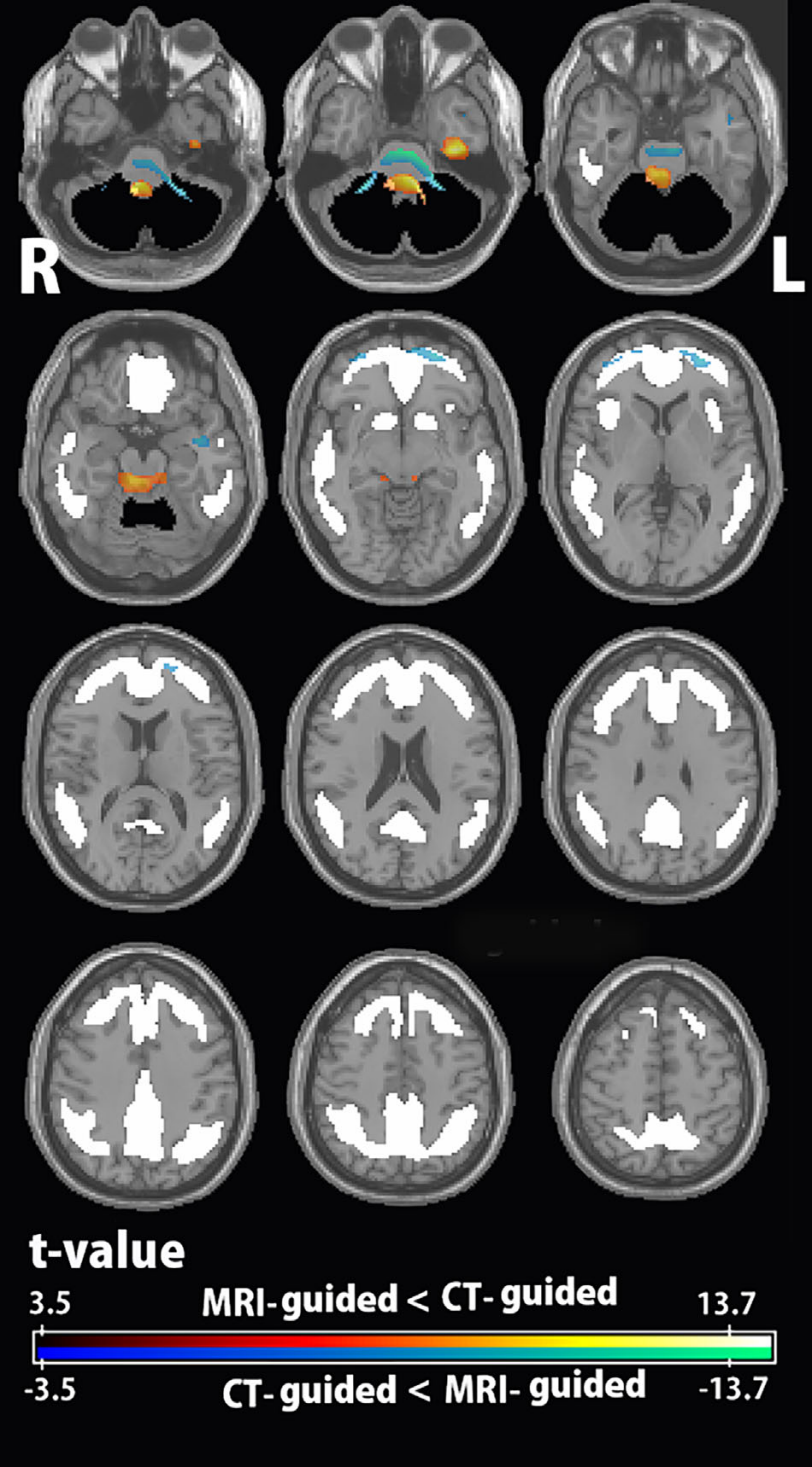
Direct comparison of anatomically standardized amyloid PET images using low‐dose CT and MRI statistical parametric mapping (SPM) analysis showed significantly (family‐wise error, *p* < .05) higher and lower uptake of CT‐guided standardized PET images than MRI‐guided standardized PET images, presented in a warm color scale and a cool color scale, respectively. Volume of interest (VOI) templates are shown as a solid black area for the whole cerebellum as a reference area and as a solid white area for the target cortical area. The largest differences in accumulation are visible in the brain stem. Lower uptake of CT‐guided standardized PET images was observed in the frontal cortex of the target cortical VOI.

Paired t‐tests performed using SPM12 (Figure 6 and Table 1) found that the brainstem exhibited the biggest differences in uptake between the CT‐guided and MRI‐guided standardized PET images. Significantly lower uptake of CT‐guided standardized PET images versus MRI‐guided PET images was observed in the frontal cortex of the target cortical VOI.

**TABLE 1:** Significant voxel‐wise differences between CT‐guided and MRI‐guided standardized PET images

	Cluster size	*T*‐Value	MNI coordinates	Location of peak voxels
	(No. of voxels)	(Peak voxel)	(*x*, *y*, *z*)	
MRI‐guided > CT‐guided	912	13.73	2, −18, −34	Ventral brain stem
	386	8.71	−26, 56, −6	Left anterior orbital gyrus
	134	8.7	−38, 0, −20	Left planum polare
	132	8.68	34, 56, −4	Right middle frontal gyrus
CT‐guided > MRI‐guided	290	13.38	−38, −14, −38	Left fusiform gyrus
	1227	11.13	−8, −38, −34	Dorsal brain stem

Abbreviation: MNI, Montreal Neurologic Institute.

## 4 DISCUSSION

In this study, we developed and validated new quantification software for amyloid PET with ^18^F‐florbetapir. This software can not only calculate the CL scale at the target cortical VOI of the GAAIN database using MRI‐guided or CT‐guided pipelines but also provide a *Z*‐score map for a participant's PET in comparison with a negative database constructed from 20 cognitively healthy adults. This software is freely available for other researchers.

The new database constructed from 20 healthy controls exhibited higher *Z*‐score values than the GAAIN database constructed from 13 healthy controls, although the GAAIN database is composed of younger subjects than the new database. This higher *Z*‐score is attributed to the lower mean cortical values of the new database compared with the GAAIN database. This new database may contribute to the earlier detection of early amyloid deposition.

Previous studies have examined the use of low‐dose CT of PET/CT equipment as a substitute for MRI in the calculation of the CL scale (Kim et al., 2022; Matsuda et al., 2021; Presotto et al., 2018). Our study using ^18^F‐flutemetamol revealed that low‐dose CT provided a CL scale comparable to that of MRI. However, the CL scale obtained with low‐dose CT was on average 1.7 points lower than that obtained with MRI. In the present study, conducted using ^18^F‐florbetapir, low‐dose CT also showed a CL scale value that was 2.1 points lower on average than that obtained with MRI. Nevertheless, the CL scale obtained with low‐dose CT was highly correlated with the gold standard CL scale obtained with MRI within the validation criteria proposed by Klunk et al. (2015). The reason why the CT_CL_ was slightly lower than the CL_MRI_ is that the CT‐guided anatomically standardized PET images showed a lower accumulation in the target cortical VOI than the MRI‐guided standardized PET images. This may be due to the slightly lower accuracy of anatomic standardization with low‐dose CT compared with MRI. On the other hand, the mean positive *Z*‐scores for the target cortical VOI compared with the new database tended to be lower for *Z*_CT_ than *Z*_MRI_ in the high range but; overall, there was no significant difference between *Z*_CT_ and *Z*_MRI_. In addition, in this regard, low‐dose CT of PET/CT equipment may be a potential substitute for MRI in amyloid PET quantification if MRI is not obtained in the same period as PET.

In contrast, several studies (Bourgeat et al., 2018; Edison et al., 2013; Fujishima & Matsuda, 2022; Imabayashi et al., 2022; Saint‐Aubert et al., 2014; Tsubaki et al., 2020), including our own research (Fujishima & Matsuda, 2022), have reported anatomic standardization using the amyloid PET template alone without MRI. Out of these studies, the uses of an adaptive template generated from a linear combination of an amyloid‐negative and amyloid‐positive template with a weight‐optimized algorithm have obtained comparable CL scales to those obtained with the standard MRI‐guided method (Bourgeat et al., 2018; Fujishima & Matsuda, 2022; Imabayashi et al., 2022). However, our correlation analysis (Fujishima & Matsuda, 2022) between this PET‐alone method and the standard MRI‐guided method showed that the CL scales slightly deviated from the validation criteria for ^18^F‐florbetapir from the Alzheimer's Disease Neuroimaging Initiative dataset (slope, 1.028; intercept, −4.302; *R* ^2^, .974). Errors in anatomic standardization may occur when the distribution of amyloid PET differs from that of the PET template, such as when there is a marked left–right difference in amyloid PET accumulation. In such cases, anatomic standardization using coregistered structural images may be more accurate than the PET‐alone method.

The limitation of this study is the small number of cases for the comparison of CL_MRI_ and CL_CT_. Further studies with more cases are needed to assess the lower limit of the dose at which the algorithm would function properly with a reduced current and whether the results would be improved by the use of diagnostic‐quality CT which increases head radiation dose to about 3 mSv. The software provides SUVR and CL scales for the entire target cortical VOI, but it may be necessary to calculate these values for segmented regions.

In conclusion, our newly developed amyloid PET quantification software for ^18^F‐florbetapir, named AMYclz, provides the CL scale and *Z*‐score to quantify the overall and local amyloid accumulation for a patient's PET compared with an amyloid‐negative database comprising 20 healthy controls. This software supports the use of low‐dose CT in PET/CT equipment, in addition to the standard use of MRI in the anatomical standardization of amyloid PET.

## CONFLICT OF INTEREST STATEMENT

Tsutomu Soma is an employee of PDRadiopharma Inc. Kyoji Okita has received a research grant from PDRadiopharma Inc. The other authors declare no conflict of interests.

### PEER REVIEW

The peer review history for this article is available at https://publons.com/publon/10.1002/brb3.3092.

## Data Availability

Research data are not shared.
